# Supplementary material for: RNA polymerase mutations cause cephalosporin resistance in clinical Neisseria gonorrhoeae isolates
Source: eLife. 2020 Feb 3;9:e51407. doi: 10.7554/eLife.51407 (PMC7012608; doi:10.7554/eLife.51407)
Supplement: Supplementary file 2. [file elife-51407-supp2.docx]

**Supplementary File 2. Ceftriaxone susceptibility and genotype of isolates that have or can acquire CRO^RS^-associated RNA polymerase mutation**

| **strain** | **CRO MIC (μg/mL)** | **CRO MIC (μg/mL) of *rpoB1* transformants** | ***ponA* (PBP1)** | **MtrR 39/45** |
| --- | --- | --- | --- | --- |
| GCGS0457 | 0.012 | 0.19 | 421P | 39A/45D |
| GCGS0092 | < 0.016 | 0.125 | 421P | 39T/45G |
| GCGS0275 | < 0.016 | 0.06 | 421L | 39A/45G |
| GCGS0336 | <0.016 | 0.25 | 421P | 39A/45D |
| GCGS0465 | <0.016 | 0.032 | 421L | 39A/45D |
| GCGS0524 | 0.012 | 0.06 | 421L | 39A/45D |
| GCGS1013 | 0.19 | n.d.^a^ | 421P | 39A/45D |
| GCGS1014 | 0.125 | n.d.^a^ | 421P | 39A/45D |
| GCGS1095 | 0.19 | n.d.^a^ | 421P | 39A/45D |
| GCGS0364 | 0.023 | n.d.^a^ | 421P | 39A/45D |

^a^ n.d., not determined. Parental isolates GCGS1013, GCGS1014, and GCGS1095 have CRO^RS^ phenotypes due to endogenous RNAP mutation; isolate GCGS0364 has not been transformed with *rpoB1*, but can spontaneously develop CRO^RS^ via *de novo* mutations in *rpoB*.
