# Supplementary material for: RNA polymerase mutations cause cephalosporin resistance in clinical Neisseria gonorrhoeae isolates
Source: eLife. 2020 Feb 3;9:e51407. doi: 10.7554/eLife.51407 (PMC7012608; doi:10.7554/eLife.51407)
Supplement: Supplementary file 3. [file elife-51407-supp3.docx]

**Supplementary File 3. Muropeptide masses detected in cell wall digests of GCGS0457 and RNAP mutants**

| **Peak** | **Proposed Structure^a^** | **Theoretical Mass (charge)** | **Observed Mass (charge)** |
| --- | --- | --- | --- |
| **1** | Tripeptide | 871.3779 (1), 436.1926 (2) | 871.3783 (1), 436.1925 (2) |
| **2** | Tetrapeptide +Ala-mDAP^2^ | 1185.5369 (1), 593.2721 (2) | 593.2719 (2) |
| **3** | Peak 2 isomer | 1185.5369 (1), 593.2721 (2) | 593.2721 (2) |
| **4** | Pentapeptide (Gly) | 999.4364 (1), 500.2219 (2) | 999.4360 (1), 500.2213 (2) |
| **5** | Tetrapeptide | 942.4150 (1), 471.7111 (2) | 942.4151 (1), 471.7109 (2) |
| **6a** | Dipeptide | 699.2931 (1), 721.275 (1+Na) | 699.2937 (1), 721.2754 (1+Na) |
| **6b** | Tripeptide (OAc) | 913.3884 (1), 457.1979 (2) | 913.3893 (1), 457.1974 |
| **7** | Pentapeptide | 1013.4521 (1), 507.2297 (2) | 1013.4527 (1), 507.2297 (2) |
| **8** | Tetrapeptide (OAc) | 984.4255 (1), 492.7164 (2) | 984.4264 (1), 492.7163 (2) |
| **9** | Pentapeptide (OAc) | 1055.4627 (1), 528.235 (2) | 1055.4684 (1), 528.2345 (2) |
| **10** | Tripeptide (–H_2_O) | 851.3517 (1), 426.1795 (2) | 851.3520 (1) |
| **11** | 4-3 tetra-tri or 3-3 tri-tetra^c^ | 897.8911 (2), 598.9299 (3) | 897.8924 (2), 598.9295 (3) |
| **12** | Peak 11 isomer | 897.8911 (2), 598.9299 (3) | 897.8924 (2), 598.9295 (3) |
| **13** | 4-3 tetra-tetra or 3-3 tri-penta^c^ | 933.4097 (2), 622.6089 (3) | 933.4117 (2), 622.6091 (3) |
| **14a** | Tetrapeptide (–H_2_O) | 922.3888 (1), 461.698 (2) | 922.3906 (1), 461.6981 (2) |
| **14b** | 4-3 tetra-penta | 968.9283 (2), 646.2879 (3) | 968.9309 (2), 646.2888 (3) |
| **15** | Pentapeptide (–H_2_O) | 993.4259 (1), 497.2166 (2) | 933.4257 (1), 497.2163 (2) |
| **16** | 4-3 tetra-tetra or 3-3 tri-penta (OAc)^c^ | 954.4150 (2), 636.6124 (3) | 954.4165 (2), 636.6124 (2) |
| **17** | 4-3 tetra-tri or 3-3 tri-tetra (–H_2_O)^c^ | 887.878 (2), 592.2544 (3) | 887.8792 (2), 592.2543 (3) |
| **18** | Peak 17 isomer | 887.878 (2), 592.2544 (3) | 887.8792 (2), 592.2543 (3) |
| **19** | 4-3 tetra-tetra or 3-3 tri-penta (–H_2_O)^c^ | 923.3966 (2), 615.9335 (3) | 923.3977 (2), 615.9332 (3) |
| **20** | Peak 19 isomer | 923.3966 (2), 615.9335 (3) | 923.3977 (2), 615.9332 (3) |

^a^ The pentapeptide stem in *N. gonorrhoeae* is L-Ala-γ-D-Glu-L-mDap-D-Ala-D-Ala. OAc = O-acetylation of MurNAc. –H_2_O = 1,6-anhdro-MurNAc. Gly = replacement of one D-Ala residue with glycine.

^b^ This structure is the product of cleavage of a 4-3 crosslink.

^c^ Mass is consistent with either a 4-3 (PBP-mediated) crosslink or a 3-3 (L,D-transpeptidase-mediated) crosslink.
