# Supplementary material for: RNA polymerase mutations cause cephalosporin resistance in clinical Neisseria gonorrhoeae isolates
Source: eLife. 2020 Feb 3;9:e51407. doi: 10.7554/eLife.51407 (PMC7012608; doi:10.7554/eLife.51407)
Supplement: Supplementary file 4. [file elife-51407-supp4.docx]

**Supplementary File 4. *N. gonorrhoeae* strains used in this study**

| Strain name | Description | Source | Strain number |
| --- | --- | --- | --- |
| GCGS0457 | CRO^S^ clinical isolate; recipient strain for transformations | GISP, CDC |  |
| GCGS1013 | CRO^RS^ clinical isolate | GISP, CDC |  |
| GCGS1014 | CRO^RS^ clinical isolate | GISP, CDC |  |
| GCGS1095 | CRO^RS^ clinical isolate | GISP, CDC |  |
| GCPH44 | CRO^RS^ clinical isolate | ([De Silva et al., 2016](#_ENREF_11)) |  |
| 28BL | CRO^S^ laboratory strain | Gift of S. Johnson |  |
| SP300-SP311 | 12 independent CRO^RS^ transformants: GCGS0457 + gDNA from GCGS1014 | This study |  |
| SP312-SP314 | 3 independent CRO^RS^ transformants:  GCGS0457 + gDNA from GCGS1095 | This study |  |
| GCGS0457 RpoD^E98K^ | Point mutation introduced on PCR product | This study | SP316 |
| GCGS0457 RpoB^R201H^ | Point mutation introduced on PCR product | This study | SP319 |
| GCGS0457 RpoD^Δ92-95^ | Deletion introduced on PCR product | This study | SP323 |
| GCGS0092 | CRO^S^ clinical isolate | GISP, CDC |  |
| GCGS0092 RpoB^R201H^ | CRO^RS^ transformant; point mutation introduced on PCR product | This study | SP349 |
| GCGS0275 | CRO^S^ clinical isolate | GISP, CDC |  |
| GCGS0275 RpoB^R201H^ | CRO^RS^ transformant; point mutation introduced on PCR product | This study | SP354 |
| GCGS0465 | CRO^S^ clinical isolate | GISP, CDC |  |
| GCGS0465 RpoB^R201H^ | CRO^RS^ transformant; point mutation introduced on PCR product | This study | SP358 |
| GCGS0336 | CRO^S^ clinical isolate | GISP, CDC |  |
| GCGS0336 RpoB^R201H^ | CRO^RS^ transformant; point mutation introduced on PCR product | This study | SP340 |
| GCGS0524 | CRO^S^ clinical isolate | GISP, CDC |  |
| GCGS0524 RpoB^R201H^ | CRO^RS^ transformant; point mutation introduced on PCR product | This study | SP368 |
| GCGS0364 | CRO^S^ clinical isolate; develops spontaneous CRO^RS^ via *rpoB* mutation *in vitro* | GISP, CDC |  |
| GCGS0364 RpoB^G158V^ | Point mutation introduced on PCR product | This study | SP377 |
| GCGS0364 RpoB^P157L^ | Point mutation introduced on PCR product | This study | SP375 |
